# The Revue Medicale and the Debate on Cancer

**Published:** 1855-02

**Authors:** 


The Revue Medicale and the Debate on Cancer.—"Let us turn a critical eye," M. Sales Girons, (disciple of M. Cayol, and editor of the organ of the Vitalists) "on that famous debate in which the School of Paris, in formal session at the Academy of Medicine, is demolishing that great edifice which cost it forty years of labor. Anatomism, like Saturn and the Revolution is at last devouring its own children.

Ninety-nine in a hundred of those who read the narrative of the present academic war, will see in it a simple scientific discussion, rather bitter it is true, about tumours in geueral, and the incurability of cancer in particular.

But the subject of tumours is only the pretext, the incurability of cancer only the outside noise of the conflict. The eternal truths of medicine are in question, and the fathers of the Anatomical School, represented by M. Velpeau, are vainly attempting to arrest the torrent which they themselves set in motion, not foreseeing whither it would lead.

When we look beneath the surface of the debate which agitates the Academy, we find the *Microscope* and the *Clinic* in mortal combat.

The microscope, an amplification of the much-vaunted scalpel, represents *matter.*

The clinic, scoffed at and disdained by the anatomo-pathologist, represents observation, *œoup d'eil*, experience, and medical tact and genius.

The microscope is the despotic instrument; the clinic is the intelligence which reasons.

The microscope is the *fact*, the clinic is the *science.*

The microscope is —— is the utter negation of medicine; the clinic is its supreme affirmation.

And yet the microscope is the very culmination, *le derniere mot* of anatomism; judge, then, anatomism. We are constantly asked to demonstrate the practical utility of sound doctrine; wait a while and look at the natural consequences of a vicious system.

Practitioners who have grown grey by the bed-side, physicians and surgeons who have earned a right to form a diagnosis, what are you thinking of? Of cancer.—Why, send a piece of the tumour to a micrographer in vogue, and by return mail you will have a ready made diagnostic, and the best prognosis the market affords.

But cancer is only the beginning; soon we shall be required to obtain the micrographer's assent to our diagnosis of phthisis; unless the sputa he inspects contains tuberculous matter our observations will be valueless; and then his domain will be extended to all morbid alterations, all normal and abnormal secretions, until medicine becomes the tributary of the microscope, the physician the servant of the micrographer.

The anatomo-pathologists decreed that matter should teach mind, that fact should predominate over science, that the senses should control the perception; here is the last step of the system,—the instrument has replaced the understanding! *patere legem quam fecisti.*

This is the true meaning of the final scene which is now being enacted at the Academy of Medicine. I say *final*, because the old or scalpel anatomists, and the young or microscope anatomists are engaged in mutual annihilation. We see the fathers struggling to avoid the abyss which they dug themselves to lay the foundations of their system, and we hear their cries.

The clinic, they exclaim through the mouth of M. Velpeau, leave us the clinic, which after all will answer for medicine and the physician. When I patronized the microscope twenty years ago, adds M. Velpeau in his own name, I was far from suspecting the pretensions which it would set up; I renounce the microscope, its pomps and vanities!

—— But admit, interrupts the microscope, that I possess the cancer cell, and consequently the diagnosis of cancer?

—— I will not admit that even, I will rather deny it, replies M. Velpeau.

—— And M. Velpeau is right; he knows the story of that child with innate diplomacy who refused to say A because he knew that afterwards he would be made to say B, and then the whole alphabet.

—— Grant us at least, resumes the microscope, the incurability of cancer?

—— Not I, says M. Velpeau, cancer is curable, I have my hand full of examples to prove it.

—— But you teach, the devil take it, urges the microscope, the pathological localization of cancer?

—— Heaven preserve me from such a doctrine, responds M. Velpeau; cancer is a diathesis, a dyscrasy, a cachexy; cancer is in the blood before it is in the breast. Cancer, interrupts M. Jules Cloquet in his place, Antoine Dubois was right about it, it is in the nervous system! (*Loud and prolonged applause from the fathers of Anatomism.*)

—— But this is the ruin of your system, cries the microscope, you are in flagrant contradiction with the principles of the celebrated school which admires you as one of its most illustrious representatives.

—— That is possible, rejoins M. Velpeau, the contradiction is my affair; I would do yet more in order to have nothing in common with you, who would destroy medicine with your radicalism. If the microscope is the termination of Anatomism, let it go, let it have its last judgment. *Solvet sæculum in favilla!*

With the aid of this little commentary, the reader will discover the true meaning of the antagonism in this academic debate. On the one hand he will perceive the microscope, respectfully aggressive; on the other clinical medicine, defended by an army of veterans, courageous rather than consistent. The micrographers are striving to maintain: 1st, the monopoly of diagnosis; 2d, the incurability of cancer; 3d, the localization of the cancerous tumour, an anatomical triad, which is a triple negation of medicine. The founders of anatomism are denying anatomical diagnosis, incurability and localization, and defending clinical observation.

And we may predict the result of the battle; the microphiles will be beaten; anatomism will be demolished, and true medicine, re-established on the ruins of a half century, will point to the conversion of M. Velpeau with more pride than to the ninety-and-nine faithful, who have adhered to her throughout the era of systems.

And will the microscope be lost? Certainly not; but clinical medicine will assign it its true place, making it subservient to order and truth; under the reign of anatomism, it could only be an instrument of anarchy."—*Med. Rev.*

